# Detection of left ventricular regional dysfunction and myocardial abnormalities using complementary cardiac magnetic resonance imaging in patients with systemic sclerosis without cardiac symptoms

**DOI:** 10.1186/1532-429X-16-S1-P337

**Published:** 2014-01-16

**Authors:** Yasuyuki Kobayashi, Masaharu Hirano, Kazuhito Nozu, Yasuo Nakajima, Hitomi Kobayashi

**Affiliations:** 1 St.Marianna University School of Medicine, Kawasaki, Kanagawa, Japan, Japan; 2 Nihon University, Tokyo, Japan; 3 Tokyo Medical Colleage, Tokyo, Japan

## Background

In patients with systemic sclerosis (SSc), myocardial involvement is common, may have serious consequences, and may lead to a poor prognosis. We aimed to detect LV regional dysfunction and myocardial abnormalities in SSc patients without cardiac symptoms using a cardiac magnetic resonance (CMR) imaging approach.

## Methods

Consecutive patients with SSc without cardiac symptoms and healthy controls underwent CMR. Peak systolic regional circumferential and radial strain (Ecc and Err, %) were calculated using a feature tracking analysis on mid-left ventricular slices obtained with cine MRI in six segments. Furthermore, perfusion defect (PD) under pharmacological stress and late gadolinium enhancement (LGE) were obtained for the assessment of myocardial abnormalities. We compared the patients and controls in terms of prevalence of CMR abnormalities, and explored possible associations between CMR abnormalities and SSc disease characteristics.

## Results

We compared 14 SSc patients with 10 healthy controls. No statistically significant differences were observed in baseline characteristics between the patients and healthy controls. The mean peak Ecc of all segments was significantly reduced in the patients compared with the controls (p = 0.004). The mean peak Err was significantly lower in the patients than the controls (p = 0.013). Five patients with LGE (28.6%) and 11 patients with PD (50.0%) were observed. The mean peak Ecc and Err in the patients with LGE was reduced compared to those without LGE, but not significant difference (p = 0.056, p = 0.077, respectively). The mean peak Ecc and Err in the patients with PD tended to be reduced compared to those without PD, but not significant difference (p = 0.250, p = 0.523, respectively). PD was significantly associated with history of digital ulcer (p = 0.005).

## Conclusions

Subclinical myocardial involvement, as detected by CMR, was prevalent in the SSc patients without cardiac symptoms.

## Funding

None.

